# A data-driven approach to identify a rapid screener for auditory processing disorder testing referrals in adults

**DOI:** 10.1038/s41598-023-40645-0

**Published:** 2023-08-21

**Authors:** Victoria E. Cancel, Jacie R. McHaney, Virginia Milne, Catherine Palmer, Aravindakshan Parthasarathy

**Affiliations:** 1 Department of Communication Science and Disorders, School of Health and Rehabilitation Sciences, University of Pittsburgh, 5060A Forbes Tower, Pittsburgh, PA 15260 USA; 2 Department of Otolaryngology, School of Medicine, University of Pittsburgh, Pittsburgh, PA USA; 3 University of Pittsburgh Medical Center, University of Pittsburgh, Pittsburgh, PA USA; 4 Department of BioEngineering, Swanson School of Engineering, University of Pittsburgh, Pittsburgh, PA USA; 5 Present Address: Department of Communication Sciences and Disorders, Northwestern University, Evanston, IL USA

**Keywords:** Auditory system, Sensory processing, Diseases, Diagnosis, Health occupations

## Abstract

Hearing thresholds form the gold standard assessment in Audiology clinics. However, ~ 10% of adult patients seeking audiological care for self-perceived hearing deficits have thresholds that are normal. Currently, a diagnostic assessment for auditory processing disorder (APD) remains one of the few viable avenues of further care for this patient population, yet there are no standard guidelines for referrals. Here, we identified tests within the APD testing battery that could provide a rapid screener to inform APD referrals in adults. We first analyzed records from the University of Pittsburgh Medical Center (UPMC) Audiology database to identify adult patients with self-perceived hearing difficulties despite normal audiometric thresholds. We then looked at the patients who were referred for APD testing. We examined test performances, correlational relationships, and classification accuracies. Patients experienced most difficulties within the dichotic domain of testing. Additionally, accuracies calculated from sensitivities and specificities revealed the words-in-noise (WIN), the Random Dichotic Digits Task (RDDT) and Quick Speech in Noise (QuickSIN) tests had the highest classification accuracies. The addition of these tests have the greatest promise as a quick screener during routine audiological assessments to help identify adult patients who may be referred for APD assessment and resulting treatment plans.

## Introduction

### Self-reported hearing difficulties despite normal audiograms

Increasing evidence suggests that a clinically normal audiogram is not sufficient to guarantee robust suprathreshold communication in complex listening environments. About 1 in 10 adult patients who seek help for hearing problems have normal hearing thresholds^1–5^. Patients with normal hearing thresholds, as defined by pure-tone thresholds ≤ 20 dB HL at octave frequencies from 250 to 8000 Hz^6^, often present to audiology clinics with primary complaints of difficulties hearing in noise and being unable to follow conversations in complex listening environments, such as in the presence of multiple speakers, reverberation, or background noise^5,7–9^. Speech perception difficulties affect a person’s productivity, personal relationships, and their overall well-being^1,10,11^. Successful speech perception involves both bottom-up sensory coding fidelity along the ascending auditory pathway^5,12–15^, as well as top-down cognitive resources such as selective attention and verbal working memory^5,7,16–19^. Hence, normal hearing thresholds on an audiogram fail to adequately capture the multidimensional processes that underlie speech comprehension under challenging listening conditions, underscoring the need for more holistic diagnostic evaluations which may support personalized treatment plans for speech perception difficulties.

### Current state of clinical practice

Currently, there is no consensus on the management of adult patients with normal audiograms who complain of hearing difficulties. Typical management strategies include counseling on effective communication strategies or low-gain hearing aids in some cases^20,21^. Clinical management for adult patients with normal audiograms is complicated by the lack of consensus on possible underlying conditions^3^, with varying terms used to refer to these conditions including central auditory processing disorder^22^, central presbycusis^23^, King–Koptezky Syndrome^24^, idiopathic discriminatory dysfunction^25^ and auditory neuropathy^26^. Additionally, recent anatomical evidence from human temporal bones and animal models suggests a loss of cochlear synapses between the inner hair cells and the auditory nerve, which is not reflected in the audiogram, and is hypothesized to cause hearing difficulties in challenging listening conditions^12,27–34^. This heterogeneity in presentation and terminology further complicates clinical management in this patient population.

### (Central) Auditory processing disorder

Of the myriad disorders attributed to hearing difficulties despite normal audiograms, APD, or central auditory processing disorder (CAPD), is arguably the most clinically recognized. APD is classified as a distinct disorder in the International Classification of Disorders manual of the World Health Organization version 10 and 11 beta version (ICD-10 and ICD-11 beta version), as well as by multiple professional associations, such as the American Speech-Language-Hearing Association^35^ and the American Academy of Audiology^36^. APD is a disorder in which there are “deficits in the neural processing of auditory information not due to higher order language or cognition”^37^. APD is characterized by poor performance in “sound localization and lateralization; auditory discrimination; auditory pattern recognition; temporal aspects of audition, including temporal integration, temporal discrimination (e.g., temporal gap detection), temporal ordering, and temporal masking; auditory performance in competing acoustic signals (including dichotic listening); and auditory performance with degraded acoustic signals”^35^. The etiology underlying APD remains under debate, but may include age-related changes, genetic determinants, neurological disorders or lesions in the central auditory pathway^38^.

Assessments for APD vary across clinics. Most APD assessments employ speech and non-speech tasks to assess potential areas of deficit within the central auditory system. Such tests can include binaural integration or separation, speech in noise, and temporal processing^39^. Binaural integration tasks are intended to test dichotic listening, lateralization, and sound localization. Testing in the binaural domain typically consists of tests where the listener is asked to verbally report stimuli heard in two conditions. In the *binaural integration* condition, the listener may be asked to report simultaneous binaural stimuli in a free recall, i.e., in which they report what was heard in either ear, in no particular order. The second condition is *binaural separation*, wherein the listener reports what was heard in a specified ear, in a directed recall^40–43^. In the speech-in-noise domain, the effects of adverse listening conditions on speech recognition are tested by presenting speech stimuli in competing levels of background noise. One example is the Quick Speech in Noise (QuickSIN) test^44^, which presents a sentence spoken in four-talker babble at six signal-to-noise ratios (SNR). Patients are asked to repeat the target sentence back to the best of their ability. The output of the QuickSIN test is a metric of SNR loss which reflects the SNR level at which the listener can correctly identify speech 50% of the time.

Within the temporal processing domain, frequency discrimination limens and gap detection are typically used to test bottom-up sensory representations of rapidly changing auditory information. These tests utilize temporal resolution, discrimination, masking, integration, and ordering during a specified time interval^45^. In addition, electrophysiological responses may be used to assess central auditory function, such as middle latency responses or late cortical responses^46^. A diagnosis of APD is typically made by the audiologist upon considering results from the combination of all of these tests, in addition to patient case histories and results from interdisciplinary assessments, such as language and cognitive assessments^36,37^.

### Referrals for APD assessment

Despite significant debates regarding the etiology, presentation, and diagnosis of APD, particularly in adult populations, an APD assessment remains one of the few clinically viable avenues of further care for adult patients with self-reported hearing difficulties despite normal audiograms. However, there are no established guidelines for referrals toward APD assessment during routine audiology visits. As more is discovered about APD, its management, and diagnoses, the question of when to provide a referral for APD assessment is critically important. There is no universally accepted method for screening for APD, and there remains a need for valid and efficient screening tools^38^. A screener for an APD referral needs to be quick to administer during routine audiometric visits and should require minimal specialized equipment that is not already present in audiology clinics.

Given the considerations discussed thus far, the current study addressed the following questions—1. What percentage of adults who sought help for self-perceived hearing difficulties at the University of Pittsburgh Medical Center (UPMC) presented with clinically normal audiograms and how many were referred for APD evaluation? 2. In the adult patients who were evaluated for APD, on which tests and domains did they demonstrate the greatest deficits? And 3. Can this information be used to propose a quick screener for use in routine audiology care to inform further referrals for APD assessment in adults? To address these questions, we analyzed large-scale patient data from eight clinics across UPMC Audiology over a 5-year period (2015–2020), and further examined the results from adult patients who underwent an APD assessment.

## Methods

### Study population

**Figure 1:**
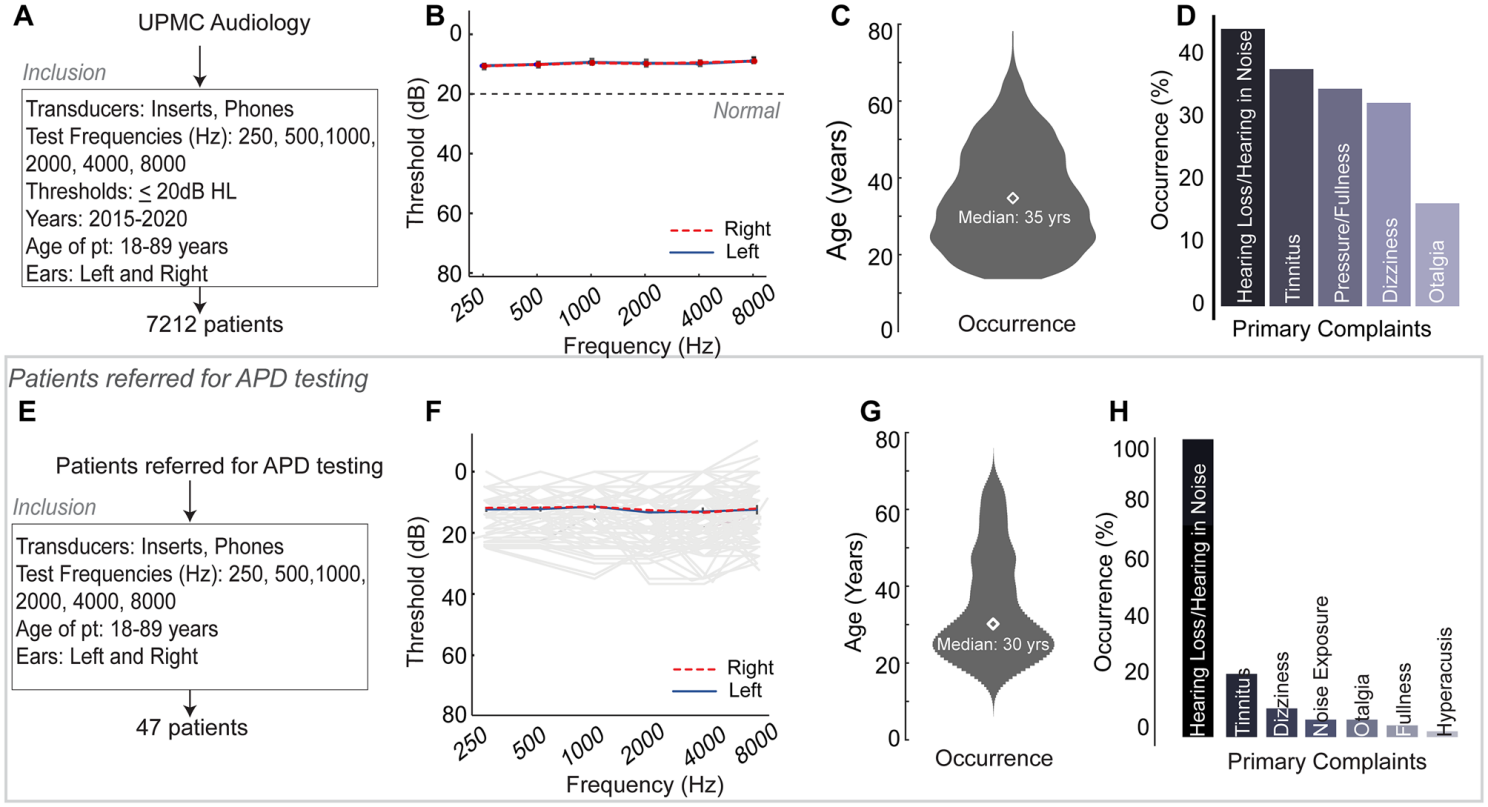
A normal audiogram does not guarantee robust hearing in everyday conditions. (**A**) Most recent patient records from UPMC audiology between 2015 and 2020 were analyzed to identify patients with bilateral normal audiograms based on the inclusion criteria listed, resulting in 7212 patients further analyzed. (**B**) Averaged hearing thresholds were better than 20 dB HL for left (blue, solid) and right (red, dashed) ears in these patients. (**C**) The median age distribution of these patients skewed towards young and middle age, with a median age of 35 years. (**D**) The primary presenting complaint for these patients was hearing loss or hearing in noise difficulties, followed by tinnitus, pressure/fullness, dizziness and otalgia. (**E**) Only 47 adult patients were further referred for APD testing. These patients also had normal or near normal bilateral audiograms (**F**), and a median age of 30 years (**G**). The primary presenting complaints in decreasing frequency were hearing loss/hearing in noise difficulties, tinnitus, dizziness, noise exposure, otalgia, fullness and hyperacusis (**H**).

We first analyzed 48,699 patient records across eight clinics affiliated with UPMC Audiology in Western Pennsylvania (Fig. 1A). Deidentified patient records were obtained using an honest broker (Health Research Records Request, University of Pittsburgh) for visits to UPMC Audiology occurring between 2015 and 2020. Deidentified patient records were obtained as comma separated value (csv) files with all sections of the audiological test battery digitized. These values and associated fields were then analyzed using scripts in R^47^. Audiometric thresholds and frequencies tested, as well as age and demographic information, were obtained from the digitized records. Patient primary complaints were extracted from clinician notes. Of the ~ 48,000 patient records, only patients who had a complete audiogram bilaterally at octave frequencies from 250 to 8000 Hz were included for further analyses (n = 47,009). Age ranges were between 18 and 89 years. We then assessed chart records of patients who were referred for and underwent further APD testing, regardless of their audiometric thresholds, though most patients had normal to near-normal audiograms. Only the most recent recorded clinic visit was analyzed for each patient, resulting in a final sample size of 47 patients. Audiometric information and primary complaints were obtained from clinician notes using the same methods noted above.

### APD test battery and patient performance

The APD test battery at UPMC consisted of three deficit domains with two tests per domain in (1) binaural integration, (2) speech-in-noise, and (3) temporal processing. In addition to the behavioral assessment, which is a routine clinical questionnaire, a screening version of the Hearing Handicap Inventory for Adults (HHIA-S)^48^ was administered to gauge subjective experiences of these patients as it related to their hearing difficulty. Additional tests as a tiebreaker were conducted for specific patients when two tests in a domain had conflicting results; however, those tests were not included in the analyses for this study, as they were not completed consistently across patients.

In the dichotic domain, the two tests administered were the Random Dichotic Digits Task (RDDT) and Dichotic Words. In RDDT, patients were presented with either one pair, two pairs, or three pairs of numbers binaurally. They were asked to repeat all digits they heard in a free recall condition, and the number of correctly recalled digits were scored per ear. A score was deemed normal if recall was greater than 90%^49^. The Dichotic Words test required the patient to repeat two pairs of words presented binaurally^50^. The patient was either asked to repeat back all the words they heard in any order, or they were instructed to report back the words heard in a specific order. The score was based on the number of words reported per ear, which resulted in the identification of a dominant and non-dominant ear. The test was scored using local normative data used at UPMC Audiology for adult patients. A normal score in the dominant ear required the patient to report 88% of the words correctly, and a normal score in the non-dominant ear required the patient to report 76% of the words correctly.

In the speech-in-noise domain, the two tests administered were the quick speech in noise (QuickSIN) test^44^ and the words-in-noise (WIN) test^51^. In QuickSIN, the patient was presented with six sentences spoken by a female voice in the presence of four-talker babble at six SNR levels: 25, 20, 15, 10, 5 and 0 dB. Each target sentence had five keywords for identification. Patients were asked to repeat the target sentence back to the best of their ability. One point was given for each keyword correctly identified. The sentences were presented to the left ear, right ear, and binaurally. The first sentence began with a signal-to-noise ratio of 25 dB. Each consecutive sentence had an SNR decrease of 5 dB, which made the task more difficult. The score was then reported as an SNR loss, which indicated the SNR level at which the patient could accurately identify words in the sentence 50% of the time^52^. A normal score for QuickSIN was an SNR loss of + 2 dB or lower, meaning the speech signal only needed to be 2 dB louder than the four-talker babble noise to achieve 50% accuracy^44^. During the WIN test, the patients were presented with lists of five words masked by varying degrees of multi-talker babble, ranging from 24 to 0 dB SNR and were instructed to repeat the words heard. Each list decreased in 4 dB steps such that each successive list of words had a lower, more-difficult SNR. The WIN score was calculated from the number of words the patient repeated correctly in varying degrees of noise. A score of 4 dB or lower was considered within normal limits, while a score of 6 dB or higher was considered two standard deviations outside of the normal range^53^.

The temporal domain was tested with the gaps in noise^45^ and frequency patterns^54^ tests. In gaps in noise, patients were presented with bursts of noise containing gaps of varying temporal durations, and the patient was instructed to indicate when they detected a gap in the noise. A normal score for gaps in noise required a patient to identify 54% or more of the gaps correctly^45^. During the frequency patterns test, patients were presented with triads of tone bursts that differed in frequency. The patient was instructed to either repeat back what they heard by specifying high pitch or low pitch, such as, ‘high low low’, or they were instructed to hum back what they heard. A normal score for Frequency Patterns required the patient to correctly identify the pattern of pitches 75% of the time or more^54,55^.

The last portion of the UPMC APD test battery was the HHIA-S in which patients responded to a subjective questionnaire about their perception of their hearing difficulties. The HHIA-S is a self-report questionnaire that assessed the patient’s reaction to their perceived hearing challenges. There were five questions on the HHIA-S that encompassed emotional reactions and five questions that assessed social/situational difficulties. Patients responded to each question with a ‘yes’, ‘sometimes’, or ‘no’, where each response was given four, two, or zero points, respectively. Possible scores on the HHIA-S ranged from 0 (no hearing handicap) to 40 (severe handicap).

Consistent with ASHA and AAA guidelines, a diagnosis of APD was made if a patient scored outside normal limits on two tests within any single domain^36,37^. If a patient scored outside normal limits on two tests that were not within the same domain, they received recommendations for auditory concerns, but not a firm diagnosis of APD. Patients who scored outside normal limits on just one test and those who scored within normal limits on all tests did not receive a diagnosis of APD.

### Statistical analysis

Variance and Pearson’s correlations were performed using standard functions (corr, var) that were used in custom written scripts in MATLAB 2020a (Mathworks). Stepwise selection was performed using a custom written script in MATLAB that used an algorithm that is described in the appropriate section in “Results”. Sensitivity, specificity, positive predictive value (PPV) and negative predictive value (NPV) and accuracy were calculated using the following formulas^56^:

$$\begin{aligned} {\text{Sensitivity}} = & \left[ {{\text{True Positive}}/\left( {{\text{True Positive}} + {\text{False Negative}}} \right)} \right] \times {1}00 \\ {\text{Specificity}} = & \left[ {{\text{True Negative}}/\left( {{\text{False Positive}} + {\text{True Negative}}} \right)} \right] \times {1}00 \\ {\text{PPV}} = & \left[ {{\text{ True Positive}}/\left( {{\text{True Positive}} + {\text{False Positive}}} \right)} \right] \times {1}00 \\ {\text{NPV}} = & \left[ {{\text{True Negative}}/\left( {{\text{False Negative}} + {\text{True Negative}}} \right)} \right] \times {1}00. \\ {\text{Accuracy}} = & {\text{Sensitivity}} \times {\text{Prevalence}} + {\text{Specificity}} \times \left( {{1} - {\text{Prevalence}}} \right) \\ \end{aligned}$$

Confidence intervals for sensitivity, specificity and accuracy are “exact” Clopper–Pearson confidence intervals^57^. Confidence intervals for predictive values are the standard logit confidence intervals^58^. Patients who received a diagnosis of APD (ICD 10 code H93.25, Central auditory processing disorder), and Auditory concerns (ICD10 code H93.29, Other abnormal auditory perceptions) were both considered a True Positive for these analyses.

This study was approved by the human subjects Institutional Review Board at the University of Pittsburgh. Requirements of informed consent were waived for this retrospective chart review by the Institutional Review Board at the University of Pittsburgh. Data analysis was performed on de-identified data, in accordance with the relevant guidelines and regulations.

## Results

### Approximately 7% of patients present to the clinic with hearing difficulties despite having normal hearing thresholds

Patient records were obtained from the UPMC database for adult patients between 18 and 89 years of age. We analyzed patient records of the most recent visit between 2015 and 2020, to identify patients with bilateral normal hearing thresholds (less than or equal to 20 dB HL) from 250 Hz through 8000 Hz in octave steps. After analyzing 47,009 patient records, we found that 7212 patients had normal hearing (Fig. 1A,B). The population of 7212 patients who had normal hearing comprised 15.34% of the total patient population who received audiograms, consistent with previously published studies^2–5^. These patients had a median age of 35 years (Fig. 1C). Among the primary complaints of the 7212 patients with normal audiograms, 3205 patients or 44% had a primary complaint related to perceived hearing loss or hearing in noise difficulties (Fig. 1D). The other primary complaints were tinnitus, pressure or fullness in the ear, dizziness, and otalgia. Note that the primary complaints were not mutually exclusive—a patient could have had more than one primary complaint. Therefore, based on the primary complaints of perceived hearing loss or of hearing in noise difficulties alone, ~ 7% of the total patient population (3205 out of 47,009) who sought help for hearing difficulties in the clinic had clinically normal hearing thresholds. These patients were provided handouts and counseling on effective communication strategies as the primary approach for managing their hearing difficulties.

### Adult APD referrals were generally very low, but the majority of patients assessed received a diagnosis consistent with APD

Only 47 patients were referred for and underwent APD testing within the UPMC system between 2015 and 2020 (Fig. 1E). The characteristics of this population closely matched those of the larger patient population with normal audiograms described above. Patients tended to have normal or near-normal audiometric thresholds (Fig. 1F) and tended to be young or middle-aged, with a median age of 30 years (Fig. 1G). These patients also reported having issues in complex listening environments. The most common complaint among all 47 patients was hearing loss and hearing in noise, followed by complaints of tinnitus, dizziness, noise exposure, otalgia, fullness, and hyperacusis (Fig. 1H).

Of the 47 patients who underwent assessment for APD, 24 scored two standard deviations outside normal limits on two tests within a single testing domain, which resulted in an APD diagnosis (ICD 10 code H93.25, Central auditory processing disorder). This was equivalent to a positive test rate of 51%. Nine patients (19.2%) tested outside normal limits on two tests in different domains, resulting in a recommendation for auditory concerns (ICD10 code H93.29, Other abnormal auditory perceptions). Three patients (8.5%) tested outside normal limits on one test alone, and 10 patients (21.3%) tested within normal limits on all tests. Patients in these last two categories did not receive an APD diagnosis nor recommendations for auditory concerns. Data from all patients who underwent the APD assessment were used for further analysis in the following sections.

### Most patients assessed for APD self-reported moderate-to-severe hearing difficulties

**Figure 2:**
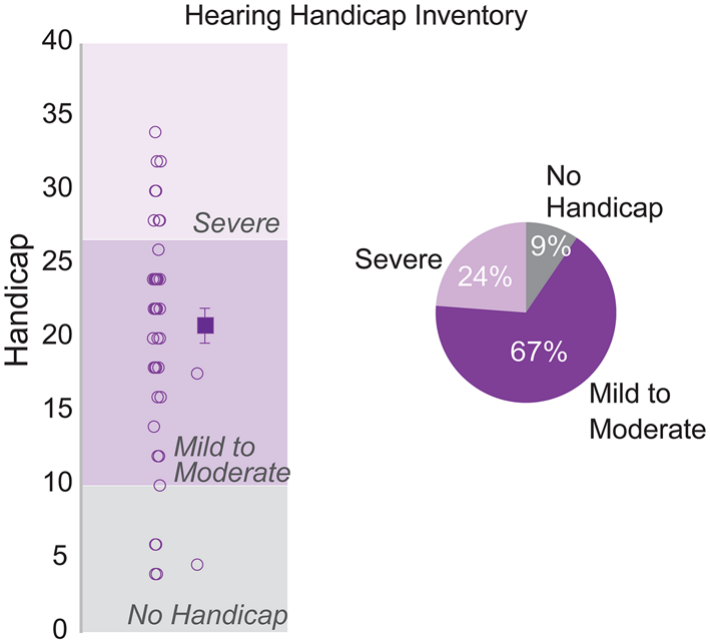
The screening version of the Hearing Handicap Inventory for Adults identifies self-reported hearing difficulties in adult patients who underwent an APD assessment. Patient self-reports on the HHIA-S classified APD patients into various degrees of hearing handicap, with most patients reporting mild to moderate hearing handicap.

The screening version of the Hearing Handicap Inventory for Adults (HHIA-S)^59^ was used as a self-assessment of hearing difficulties in patients assessed for APD. Sixty-seven percent of patients reported mild to moderate hearing handicap based on the HHIA-S, while 24% reported severe handicaps (Fig. 2). These self-reported deficits were present despite normal or near-normal audiometric thresholds, validating the primary complaints that they presented with in the clinic (Fig. 1H).

### Most deficits in adult patients undergoing APD assessment were concentrated in the binaural integration or speech-in-noise domains

We then analyzed data from the APD test battery to identify tests that consistently proved to be the most challenging for this patient population across the three domains of testing—binaural integration, speech-in-noise, and temporal processing.

**Figure 3:**
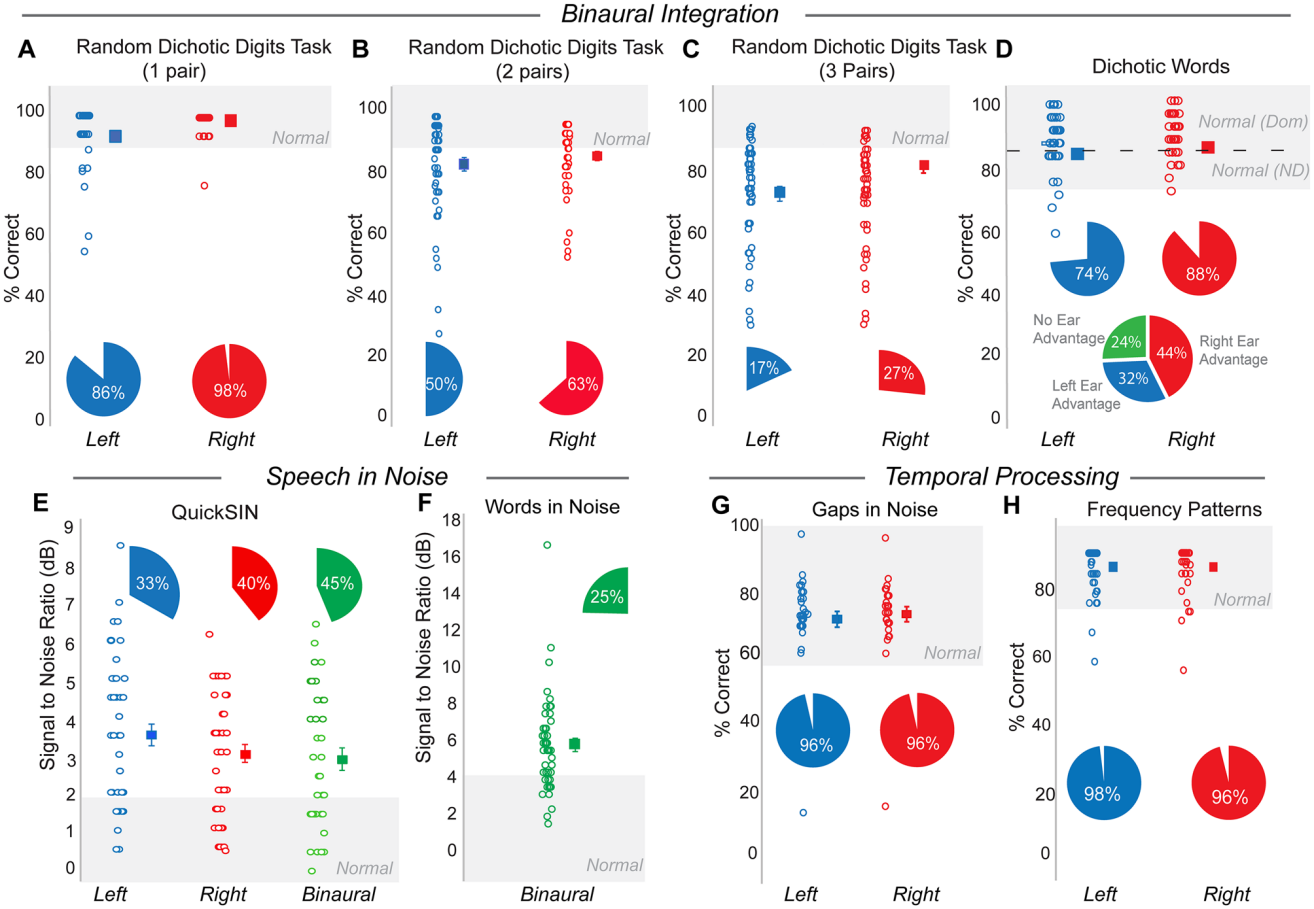
Deficits were concentrated in the binaural integration and speech-in-noise domains. Individual test data on various domains of testing are shown for the left (blue) and right (red) ears of patients assessed for APD. Shaded areas represent normal ranges for respective tests. Pie charts identify proportion of patients who tested within normal limits for each test. Tests cover the three domains of APD testing—binaural integration (**A–D**), speech in noise (**E**,**F**), and temporal processing (**G**,**H**).

The two tests administered within the binaural integration domain were the Random Dichotic Digits Task (RDDT)^49^ and the Dichotic Words task^50^. The RDDT presented the patients with either one, two, or three pairs of numbers binaurally. The patient then repeated the digits they heard and the number of correctly identified digits was scored. To receive a normal score on the RDDT, a patient must repeat back digits correctly 90% of the time. The RDDT showed decreased performance with increased task complexity, i.e., presentation of more digits (Fig. 3A–C). When one pair of digits (two digits total) was presented binaurally, 98% of patients performed normally in the right ear and 86% performed normally in the left ear. When two pairs of digits (four digits total) were presented binaurally, 63% of patients performed normally in the right ear and 50% performed normally in the left. When three pairs of digits (six digits total) were presented binaurally, 27% of patients performed normally in the right ear and 17% performed normally in the left ear.

For the Dichotic words test^50^, two pairs of words were presented binaurally, and the patients were instructed to repeat the words back. Scoring was based on the number of words correctly identified per ear, which led to the identification of a dominant ear and a non-dominant ear. Normal performance on Dichotic words is defined as correctly repeating the words 84% of the time in the dominant ear and 76% of the time in the non-dominant ear. Scores from the Dichotic words test revealed that 44% of patients had a right ear advantage, 32% had a left ear advantage, and 24% of patients had no ear advantage. Seventy-four percent of the patients performed normally in the left ear and 88% performed normally in the right ear for the dichotic words task (Fig. 3D).

Within the speech-in-noise domain, SNR loss scores from the QuickSIN test^44^ were analyzed. The QuickSIN test was comprised of six sentences masked in four-talker babble at six SNR levels from 25 to 0 dB. The SNR loss reflects the SNR level at which the listener can correctly identify speech 50% of the time. Normal performance on QuickSIN was defined by an SNR loss of + 2 dB or lower. Forty percent of patients performed normally in the right ear, 33% performed normally in the left ear, and 44% performed normally when the sentences were administered binaurally (Fig. 3E).

The other test in the speech-in-noise domain was the Words-in-Noise (WIN) test. The WIN test consisted of lists of five words accompanied by varying degrees of multi-talker babble ranging from 24 to 0 dB SNR, which decreased in 4 dB steps so that each successive list of words had a more-difficult SNR. A normal score for the WIN test was defined as a patient being able to correctly repeat words in + 4 dB SNR^53^. Twenty-five percent of patients performed normally on WIN (Fig. 3F).

Most of the adult patients in this study did not show difficulty in the temporal processing domain. Tests in the temporal processing domain included gaps in noise^45^ and frequency patterns^54^. For gaps in noise, the patient was presented with bursts of noise containing gaps of varying temporal durations. The patient was required to indicate when they heard a gap in the noise. A normal score on gaps in noise was defined as correctly identifying the gaps 54% of the time. Ninety-six percent of patients were able to do this in both the left and right ear (Fig. 3G).

The frequency patterns^54^ test presented patients with triads of tone bursts that differed in frequency. The patient then either repeated back what was heard by specifying high pitch or low pitch such as, ‘high low low’, or they were required to hum back what was heard. A normal score on the frequency patterns test was defined as correctly repeating the triad of tone bursts 75% of the time^54,55^. Ninety-six percent of patients were able to do this in the right ear and 97% in the left ear (Fig. 3H).

### WIN, RDDT, and QuickSIN identify most patients with APD

**Figure 4:**
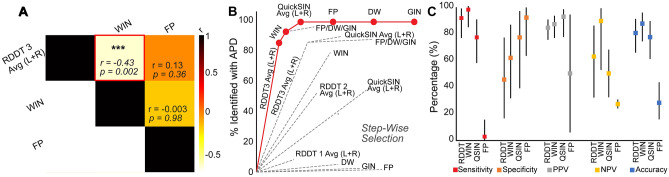
Development of a rapid screener for APD referrals. (**A**) Across domains, only performance on the RDDT 3-pair test and the words-in-noise test were significantly correlated in patients assessed for APD. (**B**) Stepwise selection revealed the shortest path for capturing the greatest number of deficits in patients assessed for APD (red, solid) compared to all other combinations of tests (gray, dashed). The cumulative increase in hit rate with each successive addition of a test is shown on the y axis. *RDDT* random dichotic digits test, *GIN* gaps in noise test, *FP* frequency patterns test, *QuickSIN* quick speech in noise, *DW* Dichotic words test, *WIN* words-in-noise test. (**C**) Sensitivity, specificity, positive predictive value (PPV), negative predictive value (NPV) and Accuracy for the top three tests identified via stepwise selection (RDDT, WIN, QuickSIN) contrasted with the lowest test (FP). Error bars indicate 95% confidence intervals.

Pearson's correlations were used to assess the relationship between the most challenging test in each of the three auditory processing domains—RDDT 3pair, WIN and frequency patterns—and to identify a potential combination of tests for a screening test battery. The RDDT three pair condition within the binaural integration domain and WIN in the speech-in-noise domain were significantly correlated (r = − 0.43, *p* = 0.002). No significant correlations were observed within the temporal domain (Fig. 4A).

Next, a stepwise selection was performed to settle on the smallest subset of tests that accounted for the largest proportion of patients within this population who performed below normal. The entire test battery was analyzed to identify the test that had the largest proportion of patients whose results fell outside the normal range. In the next step, every remaining test was then systematically added to the first identified test, and the resultant hit rate was determined. This process was repeated for every subsequent combination of tests. If two tests or combinations of tests produced the same hit rate, they were both selected, and the next selection step repeated. In this manner, every possible combination of the tests were analyzed to determine the likelihood that a patient would fail at least one test with the least number of steps. The optimal test set is shown in red in Fig. 4B. The three pair condition of RDDT alone resulted in 83% of the patients testing outside normal limits. Adding words-in-noise increased the percentage of patients testing outside normal limits to 93%. Adding QuickSIN provided a modest increase to 97%. Addition of subsequent tests did not further increase the number of patients testing outside normal limits.

Finally, based on the stepwise selection, the sensitivity, specificity, positive predictive value, negative predictive value, and accuracy were calculated for the three most predictive tests identified: RDDT (3-pair), WIN and QuickSIN. These values were then contrasted with values obtained for frequency processing (the lowest scoring test in the stepwise selection) using the equations described in the methods. Calculations for sensitivity, specificity, positive predictive value, negative predictive value, and accuracy used the results from all 47 patients assessed for APD. For the purposes of these analyses, both patients who received a diagnosis of APD as well as those who received a diagnosis of auditory concerns by the audiologist were considered a true positive. These results, along with 95% confidence intervals, are shown in Fig. 4C. Sensitivity, i.e., the probability that a test result would be positive when APD was present, was highest for WIN (96.9%), followed by RDDT 3-pair (91.2%) and QuickSIN (76.67%). In contrast, FP only had a sensitivity of 3%. Specificity, i.e., the probability that a test result would be negative when a diagnosis of APD was not made, was 61.5% for WIN, followed by 45.54% for RDDT, 77.77% for QuickSIN and 92.3% for FP. The positive predictive value (PPV), i.e., the probability of APD being present when the test is positive, was highest for QuickSIN at 92%, followed by WIN (86.5%) and RDDT (83.8%). In contrast the PPV for FP was only 50%. The negative predictive value (NPV), i.e., the probability that APD was not present, when the test was negative, was 88.9% for WIN, followed by RDDT (62.5%), QuickSIN (50%) and FP (27.3%). Finally, accuracy, i.e., the overall probability that a patient was correctly classified, was highest for WIN (87%), followed by RDDT (80%) and QuickSIN (76.9%), as opposed to 28.3% for FP.

## Discussion

### A case for increased referrals for APD testing

Increasing evidence demonstrates that a normal audiogram is not enough to guarantee robust hearing in complex listening environments. Here, we analyzed 48,699 audiograms from a 5-year period from UPMC audiology clinics to identify adult patients who may fit this profile. Our clinical data showed that approximately 15% of UPMC adult patients had normal audiometric thresholds, wherein 7% had some form of perceived hearing deficit (Fig. 1). These numbers aligned closely with previously published studies, which suggest that 5–10% of patients coming to the clinic have some form of perceived hearing deficits despite normal audiograms^2,3,5^. While many of these patients may have been good candidates for APD assessment referral, the guidelines for referring a patient to APD testing, and the APD test battery itself, varies among practices. Within the UPMC clinic database, only a small number of adult patients were referred for and further underwent APD testing, though many more may have been eligible for referral (Fig. 1). The majority of patients who were referred and underwent further testing for APD obtained a diagnosis of APD or auditory concerns. However, the lack of a *gold standard* for APD referrals makes appropriate referrals that lead to APD diagnosis and resultant treatment plans challenging.

There is a critical need for increased education surrounding the clinical indicators of APD. The ability to identify a potential case of APD based on case history, self-assessments, and audiometric testing will increase referrals and diagnoses, leading to more patients receiving treatment for APD. An interview of the patient and any present family members to gain information about their everyday experiences with their hearing difficulties also helps to determine APD referrals^37,38^. Subjective questionnaires, such as the HHIA-S^59^, also help to quantify adverse listening experiences patients face. Finally, there exists a need for valid and efficient screening tools for APD^38^.

### A rapid screener to inform APD referrals

In addition to patient reports and self-description of hearing difficulties, the inclusion of a few additional tests may be useful as a rapid screener to inform APD referrals for adult patients. When patient performance on UPMC’s test battery was analyzed, dichotic listening was the most difficult condition for the assessed patients (Fig. 3A–C), particularly when task difficulty increased. Following dichotic listening, speech-in-noise was the most challenging domain for patients assessed for APD (Fig. 3E,F). Temporal processing was largely normal for the adult patients in this study (Fig. 3G,H), which involved detection of gaps-in-noise and frequency discrimination, both relatively simple tests of temporal fidelity. More complex temporal processing tests might reveal further deficits in patients with APD, as evidenced by the QuickSIN and RDDT results, which, along with their specific domain, also test for temporal integration of complex information that form an auditory stream^44,49^.

Performance on the words-in-noise and the RDDT tests were significantly correlated in patients assessed for APD, suggesting shared underlying mechanisms that may contribute to these deficits (Fig. 4A). Patients performed slightly better in their right ear compared to the left. Many individuals tend to perceive stimuli presented to their right ear more accurately than the left ear during dichotic listening, due to the right ear advantage^60,61^. Right ear advantage is thought to occur because the left ipsilateral pathway shows an increased rate of inhibition compared to the right ipsilateral pathway, especially when stimuli are presented simultaneously^60,61^.

A stepwise selection was then performed to determine the tests that patients who underwent APD assessment found the most challenging. RDDT, followed by words-in-noise and QuickSIN resulted in the greatest hit-rate in identifying perceptual deficits in patients assessed for APD. Based on this selection model, these three tests were chosen to calculate sensitivity, specificity, PPV, NPV and accuracy, and these values were contrasted with FP, which showed the lowest score in the stepwise selection (Fig. 4B). Based on this analysis, all three tests were identified as a reasonable screener to inform APD referrals, with each test differing slightly in terms of these metrics (Fig. 4C). Overall, WIN performed the best, having the highest accuracy, sensitivity and NPV in terms of detecting patients with a diagnosis of APD or other auditory concerns. QuickSIN had the highest PPV, though it had a lower sensitivity and NPV. RDDT had a reasonable sensitivity, PPV and accuracy, though the specificity was low. Ultimately, any combination of these tests can be a reasonable screener to inform referrals for APD assessment. The actual make-up of this screener can be customized based on tolerances, available time, equipment, and testing expertise in an individual clinic. If time constraints dictate the use of one test alone, then our data suggests that the WIN test can be effectively used for APD screening to support appropriate referrals.

### Caveats and limitations

Certain caveats need to be considered when interpreting the results of this study. While a large number of patients presented with self-reported hearing loss or hearing in noise difficulties despite normal audiograms (n = 3205), the number of patients who were referred for APD assessment was very low (n = 47; Fig. 1). Most other patients who were not referred for APD assessment received counseling and a handout on effective communication strategies. The lack of a standardized APD referral process and the low referral numbers were, in part, the motivation for this study. Hence, while this study should be considered an exploratory analysis using a limited data set on trends seen in APD testing, these trends can inform referral practices for increased APD assessment.

In the absence of standardized treatment options for APD, a valid concern is whether there is a need for increased APD referrals in the first place, given the same treatment options may be provided based on routine audiometry and patient self-report of hearing difficulties. However, in current clinical practice, APD assessment is much more in depth, taking up to 60–120 min, as opposed to a standard audiological visit, which takes 15–30 min. In addition to the validation of hearing concerns, management of APD typically includes extensive counseling measures to ensure the patient understands the condition they have and to instill realistic expectations. The patient can be informed that their neural processing may be disrupted, which can cause challenges in complex listening environments.

Most treatment options for APD focus on improving the signal-to-noise ratio to support successful communication. This may include counseling on effective communication strategies such as facing the individual who is speaking, positioning themselves near the speaker, moving to quieter spaces for communication or the use of remote microphone technology to improve the signal-to-noise ratio in challenging communication environments. In addition, supplementing the auditory signal with text (e.g., speech to text in live conversation, media captioning, phone captioning, etc.) may be recommended. Other management options include low-gain hearing amplification, The proliferation of over the counter (OTC) hearing aids including self-fitted amplification methods may prove to be valuable as an alternate pathway to obtain low-gain amplification in this patient population^62,63^ though further research is needed to determine the viability of OTC hearing aids as an effective treatment option for APD. Finally, management can include aural rehabilitation, such as training in challenging listening environments, including practice with environmental manipulations and technology solutions. Patients also can complete listening exercises aimed to promote confidence in challenging listening situations. Patients may get further referrals for neuropsychiatric evaluation or neurology consults as necessary. Finally, they may receive educational, or employment accommodations as needed based on these results. A recommendation for accommodations would need to be based on metrics indicating difficulty in specific domains as opposed to self-report alone. These approaches have the ability to significantly improve quality of life^1,35–37^. Additionally, as the next generation of objective diagnostic tests become available for APD assessment^64^, the use of a screener for APD referrals will become more valuable.

Finally, while sensitivity and specificity can be calculated with the study sample along with 95% confidence intervals, PPV, NPV, and accuracy assume disease prevalence based on our limited sample size. This is another caveat that needs to be considered when interpreting these results.

### Summary and conclusions

A large proportion of patients seeking help for hearing difficulties have normal audiometric thresholds. Currently, the most viable clinical option for further management is assessment for APD, but there are no published guidelines for screening techniques that would lead to appropriate APD test referral. In the current study, we analyzed test results from the APD assessments at UPMC to propose screening tools to inform future APD referrals. Based on our results, WIN, QuickSIN, or RDDT may be useful screener tools to motivate a referral for comprehensive APD testing. The exact screener test can be individualized based on tolerances for specificities and accuracies, as well as constraints related to time and equipment.

## Data Availability

All relevant data are within the paper. Raw data that support the findings of this study are available from the corresponding author, upon reasonable request.
